# Single-Cell Atlas Reveals Complexity of the Immunosuppressive Microenvironment of Initial and Recurrent Glioblastoma

**DOI:** 10.3389/fimmu.2020.00835

**Published:** 2020-05-07

**Authors:** Weilun Fu, Wenjing Wang, Hao Li, Yuming Jiao, Ran Huo, Zihan Yan, Jie Wang, Shuo Wang, Jiangfei Wang, Dexi Chen, Yong Cao, Jizong Zhao

**Affiliations:** ^1^Department of Neurosurgery, Beijing Tiantan Hospital, Capital Medical University, Beijing, China; ^2^China National Clinical Research Center for Neurological Diseases, Beijing, China; ^3^Institute of Hepatology, Capital Medical University Affiliated Beijing You’an Hospital, Beijing, China

**Keywords:** glioblastoma, recurrent glioblastoma, CyTOF, immune profiling, microenvironment

## Abstract

The Glioblastoma (GBM) immune microenvironment plays a critical role in tumor development, progression, and prognosis. A comprehensive understanding of the intricate milieu and its interactions remains unclear, and single-cell analysis is crucially needed. Leveraging mass cytometry (CyTOF), we analyzed immunocytes from 13 initial and three recurrent GBM samples and their matched peripheral blood mononuclear cells (pPBMCs). Using a panel of 30 markers, we provide a high-dimensional view of the complex GBM immune microenvironment. Hematoxylin and eosin staining and polychromatic immunofluorescence were used for verification of the key findings. In the initial and recurrent GBMs, glioma-associated microglia/macrophages (GAMs) constituted 59.05 and 27.87% of the immunocytes, respectively; programmed cell death-ligand 1 (PD-L1), T cell immunoglobulin domain and mucin domain-3 (TIM-3), lymphocyte activation gene-3 (LAG-3), interleukin-10 (IL-10) and transforming growth factor-β (TGFβ) demonstrated different expression levels in the GAMs among the patients. GAMs could be subdivided into different subgroups with different phenotypes. Both the exhausted T cell and regulatory T (Treg) cell percentages were significantly higher in tumors than in pPBMCs. The natural killer (NK) cells that infiltrated into the tumor lesions expressed higher levels of CXC chemokine receptor 3 (CXCR3), as these cells expressed lower levels of interferon-γ (IFNγ). The immune microenvironment in the initial and recurrent GBMs displayed similar suppressive changes. Our study confirmed that GAMs, as the dominant infiltrating immunocytes, present great inter- and intra-tumoral heterogeneity and that GAMs, increased exhausted T cells, infiltrating Tregs, and nonfunctional NK cells contribute to local immune suppressive characteristics. Recurrent GBMs share similar immune signatures with the initial GBMs except the proportion of GAMs decreases.

## Introduction

Glioblastoma (GBM) is the most common and aggressive primary brain tumor (1). Because of their malignant growth and invasion into the brain parenchyma coupled with resistance to therapy, GBMs are among the deadliest of all tumors (2). A recent study demonstrated that through hijacking the immune system, GBM cells limit the efficacy of conventional therapies (3). GBM cells secrete numerous factors that promote tumor infiltration of a range of immunocytes (4, 5). Locally produced factors and their crosstalk with the extracellular matrix drive and reprogram infiltrating immune cells to acquire distinct functional phenotypes (6). These infiltrating immune cells have been shown to engage in reciprocal interactions with neoplastic tumor cells to play a crucial role in tumor growth, metastasis, and response to treatment (7).

Glioma-associated microglia/macrophages and tumor-infiltrating lymphocytes constitute the major infiltrating immune cell population (8, 9). Microglia and peripheral macrophages, which extensively infiltrate GBMs, are collectively termed GAMs (10); tumor-infiltrating lymphocytes mostly comprise CD4+ T cells, CD8+ T cells, and regulatory T cells (Tregs) (4). Current checkpoint blockade therapies mainly function to rescue T cells from exhaustion or to deplete Tregs, while GAM-targeted treatments may improve the prognosis of GBM patients as immunotherapeutic interventions (11). Dissecting the details of immune cells, particularly regarding GAMs and T cell function and distribution at tumor sites, might lead to novel strategies to further strengthen anti-tumor immunity.

Compared with initial GBM, recurrent GBM is thought to exhibit different clinical features, molecular subtypes and gene alterations (12, 13). Preliminary data suggest that neoadjuvant anti-programmed cell death protein 1 (PD-1) immunotherapy promotes a survival benefit with intra-tumoral and systemic immune responses for patients with recurrent GBM (14). While several trials utilizing anti-PD-1 or anti-programmed cell death-ligand 1 (PD-L1) are currently ongoing in patients with initial GBM (15), whether the specific features of recurrent GBM create unique immune changes and exhibit differences in their immune microenvironment remain unclear.

Developing immunotherapies that are effective against initial or recurrent GBM requires combinatorial strategies that target multiple aspects of immune tolerance (16). Realizing this potential requires a comprehensive understanding of the GBM immune microenvironment. In this study, we applied a single-cell level technology mass cytometry (CyTOF) (17) to capture immunocyte populations in situ to determine their roles in both the microenvironment and the peripheral blood and to address the cellular and molecular complexity of the immunosuppressive microenvironment in initial and recurrent GBM. Our data provide a detailed dissection of GBM immune cell types, revealing inter- and intra- tumoral heterogeneity of GAMs and T cell exhaustion in GBM lesions. These observations will facilitate a better understanding of the complexity of the immunosuppressive microenvironment of initial and recurrent GBM and will benefit in designing patient-specific immunotherapy.

## Materials and Methods

### Human Specimens and Ethics Statements

Blood and GBM tissues were obtained from GBM patients undergoing craniotomy surgery at Beijing Tiantan Hospital (Beijing, China) from 2018.7 to 2018.10 after informed consent was provided. All cases were confirmed by histopathology. Healthy donor peripheral blood was taken from healthy volunteers after obtaining informed consent. None of the patients or healthy donors used glucocorticoids before sampling. This research was approved by the Institutional Review Board (IRB) and Ethics Committee of Beijing Tiantan Hospital (Beijing, China). Written informed consent was obtained from all patients and healthy donors.

### GBM Tissue Single-Cell Dissociation

Glioblastoma tissues were washed with ice-cold Dulbecco’s phosphate-buffered saline (DPBS, without Mg^2+^ and Ca^2+^, catalog no. D8537, Sigma-Aldrich, St. Louis, MO, United States) immediately after the operation. Briefly, the samples were dissociated using type IV collagenase (catalog no. 17104019, GIBCO, Gaithersburg, MD, United States) for 10 min at 37°C. Then, the samples were washed with Dulbecco’s Modified Eagle’s Medium (DMEM, catalog no. D5796, Sigma-Aldrich, St. Louis, MO, United States) and centrifuged (4 min at 300 *g*, 18°C, minimal braking). The samples were then filtered through a 70 mm cell strainer with DPBS and washed with red blood cell (RBC) lysis buffer (catalog no. 555899, BD Biosciences, Franklin Lakes, NJ, United States). The dissociated cell suspension was then washed once with DPBS. The cell pellet was resuspended in 1 mL of staining buffer (DPBS containing 5% fetal bovine serum, FBS; catalog no. 0500, ScienCell, Carlsbad, CA, United States) and washed one more time.

### Blood Single-Cell Dissociation

Fresh blood samples were collected into ethylenediaminetetraacetic acid (EDTA) anticoagulation tubes and then centrifuged (5 min at 800 *g* with minimal braking) to remove plasma. Then, the samples were transferred into SepMate PBMC isolation tubes containing Ficoll (catalog no. 86450, STEMCELL Technologies, Vancouver, Canada) and centrifuged (10 min at 1200 *g*, minimal braking). The cells were washed with RBC lysis buffer and then washed twice with staining buffer.

### Mass Cytometry

A panel of 30 antibodies designed to distinguish a broad range of immune cells was used. Antibodies were either purchased in a preconjugated form from Fluidigm (South San Francisco, CA, United States) or purchased from Biolegend (San Diego, CA, United States) in a purified form and conjugated in-house using the Maxpar X8 Multimetal Labeling Kit (catalog no. 201300, Fluidigm, South San Francisco, CA, United States) according to the manufacturer’s instructions. The antibodies and reporter isotopes are included in Supplementary Table S1. Briefly, cell samples were rapidly rewarmed and then washed and stained with cell surface antibodies for 30 min on ice. Subsequently, the samples were permeabilized at 4°C overnight and stained with intracellular antibodies for 30 min on ice. The antibody-labeled samples were washed and incubated in 0.125 nM intercalator-Ir (catalog no. 201192B, Fluidigm, South San Francisco, CA, United States) diluted in phosphate-buffered saline (PBS, catalog no. 806544, Sigma-Aldrich, St. Louis, MO, United States) containing 2% formaldehyde and stored at 4°C until mass cytometry examination. Before acquisition, the samples were washed with deionized water and then resuspended at a concentration of 1 × 10^6^ cells/mL in deionized water containing a 1:20 dilution of EQ Four Element Beads (catalog no. 201078, Fluidigm, South San Francisco, CA, United States). The samples were then examined by CyTOF2 mass cytometry (Fluidigm, South San Francisco, CA, United States).

### CyTOF Data Analysis

Data were obtained in the form of .fcs files. The addition of EQ Four Element Beads allowed us to use the MATLAB-based normalization technique using bead intensities as previously described (18). The CyTOF data were analyzed on Cytobank^1^. Cell types were identified based on the following parameters: T cells, CD45+CD3+; B cells, CD45+CD19+; natural killer (NK) cells, CD45+CD3-CD16+CD56+ (19); monocytes, CD45+CD14+CD16+ (20); macrophages or microglia cells, CD45+CD11b+CD68+ (21); Tregs, CD45+CD4+CD25+CD127- (22); naïve CD4+ T cells, CD45+CD45RA+CCR7+CD4+ (23); and naïve CD8+ T cells, CD45+CD45RA+CCR7+CD8+ (23). Monocytes and macrophages constitute mononuclear phagocytes (24) (Supplementary Table S2). Manual gating was applied to mark cell types as previously reported (25). Data were analyzed using viSNE (26) algorithms on the indicated gated cells. Then, automatic cluster gate functionality was used for the hierarchical cluster analysis. The number of events to be sampled was set by the maximum available cell numbers in the smallest sample to avoid skewing the data toward larger samples. Heatmaps of marker expression and relative marker expression were generated by R software (version 3.4.0).

### Heatmap Data Normalization

For Figure 3A, we compared the indicated factor expression level of GAMs to that of each paired patient peripheral blood mononuclear cells (pPBMC) sample (1:1 comparison). The relative factor expression level of GAMs was obtained by calculating the log10-normalized value of the ratio of the mean expression level of the factor in GAMs over its expression level in the paired pPBMC sample. For each factor i in patient j, the formula is summarized as follows:

$$R\,e\,l\,a\,t\,i\,v\,e\,e\,x\,p\,r\,e\,s\,s\,i\,o\,n\,l\,e\,v\,e\,l\,E_{i\,j}=l\,o\,g\,10\,\left(\bar{E_{G\,A\,M_{i\,j}}}/\bar{E_{p\,P\,B\,M\,C_{i\,j}}}\right)$$

E is the relative expression level.

For Figures 3C, 4B, 5G, the log10-scaled values were first used to normalize the mean value of each marker, and min-max normalization was then used to obtain the final normalized values in the heatmap. The min-max normalization formula is as follows:

$$z=\frac{x-m\,i\,n\,\left(x\right)}{\max\left(x\right)-m\,i\,n\,\left(x\right)}$$

Z is the final normalized value, x is log10-scaled value; and min and max are the minimum and maximum log10-scaled values, respectively.

### Histology and Immunofluorescence Staining

Glioblastoma samples were fixed overnight in 4% formalin (4°C) and embedded in paraffin blocks for paraffin sections. Hematoxylin and eosin (H&E) staining was performed as previously described (27). For immunofluorescence, paraffin sections (3 μm) were washed twice 15 min in PBS (catalog no. 806544, Sigma-Aldrich, St. Louis, MO, United States), permeabilized in 0.2%–0.5% Triton X-100 (catalog no. T8200-100, Solarbio, Beijing, China) and blocked in 5% normal donkey serum (catalog no. 017-000-001, Jackson Lab, West Grove, PA, United States) for 1 h and stained with primary antibody overnight. Primary antibody were detected using fluorescent-conjected second antibodies (catalog no. PV-6000, ZSGB-BIO, Beijing, China). Sections were mounted with fluorescence mounting medium (catalog no. S3023, Dako, Glostrup, Denmark). As previously described (28), the Opal 4-Color Manual IHC Kit (catalog no. NEL810001KT, Perkin Elmer, Waltham, MA, United States) was used for the analysis of formalin-fixed paraffin-embedded GBM sections according to the manufacturer’s protocol. Fluorescent images were acquired on a Zeiss LSM880 NLO microscope and Zeiss Axio Scope A1 was used to obtain H&E images. Primary antibodies were: anti-CD45 (catalog no. AB40763, Abcam, Cambridge, MA, United States), anti-CD68 (catalog no. AB955, Abcam, Cambridge, MA, United States), anti- tumor necrosis factor α (TNFα) (catalog no. 60291-1-Ig, Proteintech, Rosemont, IL, United States) and anti-indoleamine-pyrrole 2, 3-dioxygenase (IDO) (catalog no. 86630S, CST, Danvers, MA, United States).

### Statistics

For CyTOF experiments, 13 initial GBM samples and nine paired blood samples, three recurrent GBM samples and three paired blood samples, and eight healthy donor blood samples were analyzed. For comparison of the nine initial GBM tissues and paired pPBMCs, the Wilcoxon matched-pairs signed rank test was used. Additionally, for the initial GBM tissues samples, recurrent GBM tissue samples and hPBMCs, the Mann–Whitney test was used to analyze each cell subset. Statistical analysis was performed using GraphPad Prism 7.00 software. *P*-values less than 0.05 were considered statistically significant.

## Results

### Single-Cell Profiling of the GBM Immune Microenvironment

We obtained 13 initial GBM tumor tissues, nine of which had paired pPBMC samples. The blood samples of the other 4 initial cases didn’t pass quality control for CyTOF test. We also obtained three additional recurrent GBM tumor tissues and three paired pPBMC samples. The baseline characteristics of all patients are summarized in Table 1. Eight healthy donors provided peripheral blood samples (hPBMCs) as a control.

**TABLE 1:** Basic characteristics of all 16 patients.

**Variable**	**Initial GBM (*N* = 13)**	**Recurrent GBM (*N* = 3)**
Age-mean, years (range)	55.5(31−74)	45.5(36−63)
Male	10(76.9%)	1(33.3%)
Female	3(23.1%)	2(66.6%)
IDH1		
Mutation	4(30.8%)	1(33.3%)
Wild type	9(69.2%)	2(66.6%)
IDH2		
Mutation	0(0%)	0(0%)
Wild type	13(100%)	3(100%)
TERT promoter		
C228T	3(23.1%)	1(33.3%)
C250T	4(30.8%)	0(0%)
Wild type	6(46.2%)	2(66.6%)

*N, number; IDH, isocitrate dehydrogenase; TERT, telomerase reverse transcriptase.*

**FIGURE 1:**
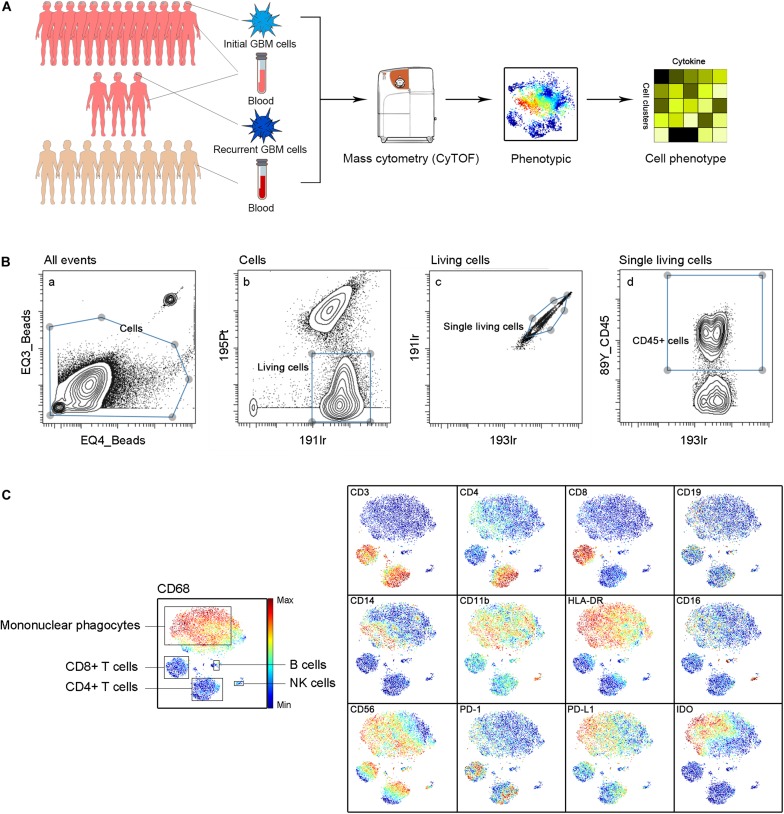
Immunosuppressive microenvironment of initial and recurrent GBM using CyTOF. **(A)** Schematic for defining the immune composition of GBMs. Initial or recurrent tumor tissues and pPBMCs were collected from patients, and hPBMCs were collected from healthy donors. Samples were processed and stained with antibodies conjugated to metal isotopes. CyTOF single-cell data were used to identify the immune features of patients. **(B)** All ungated events were sequentially gated in Cytobank to identify living single cells. (a) 151Eu EQ3 and 153Eu EQ4 beads were used to identify cells. (b) Living cells were identified by gating cells negative for 195Pt. (c) 191Ir and 193Ir were used to obtain living single cells from (b). (d) CD45+ cells were obtained from living single cells. **(C)** ViSNE analysis of immune cells from samples indicated by the relative expression of CyTOF markers for a representative patient; the cell populations are also indicated (left).

Approximately 37000 CD45+ cells were detected on average per sample. We simultaneously mapped the immune compartments of GBM lesions and pPBMCs. We also compared pPBMCs with hPBMCs to distinguish changes in GBM circulating immunity (Figure 1A). The initial gating strategies for the single living cells are provided in Figure 1B. The dimensionality reduction tool viSNE (26) was employed to convert high-dimensional CyTOF data from each sample into a two-dimensional map (Figure 1C).

### Mononuclear Phagocytes and T Lymphocytes Dominate the Initial GBM Microenvironment

**FIGURE 2:**
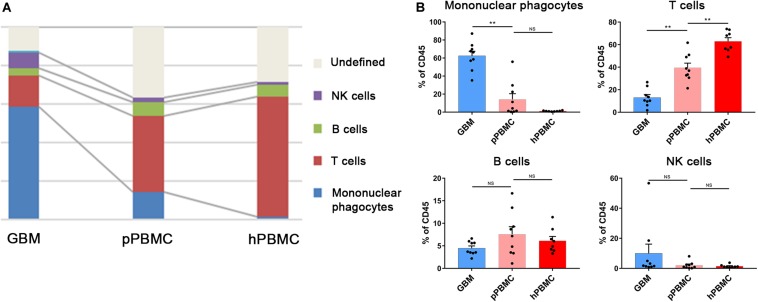
Immunosuppressive changes in the initial GBM microenvironment and circulating immunity. **(A)** Composition of the CD45+ compartment showing the average frequencies of major immune lineages for each tissue. **(B)** Bar plots showing the frequencies for each initial patient and pPBMC sample (by Wilcoxon matched-pairs signed rank test) and the frequencies for each pPBMC and hPBMC sample (by the Mann–Whitney test). Bar plots show the mean with SEM (NS, no significance; **p < 0.01).

We analyzed the distributions of the different immune cell lineages that accumulated in initial GBM lesions and pPBMCs across patients. The most abundant immune cells in initial GBM lesions were mononuclear phagocytes (59.05%) and T lymphocytes (16.39%). Compared with that in pPBMCs, the proportion of mononuclear phagocytes at the tumor site was significantly increased (*p* < 0.01), while the proportion of T cells was significantly decreased (*p* < 0.01) (Figures 2A,B). The remaining CD45+ cells constituted immunocytes that could not be defined by markers in this panel.

To investigate changes in the circulating immunity of GBM patients, we also compared PBMCs from GBM patients and healthy donors. The results showed a diminished T cell fraction in GBM patient peripheral blood compared to that in healthy donors (*p* < 0.01), while the proportions of NK cells and B cells were similar across all samples (Figures 2A,B).

### Diversity of GAM Subsets in GBM Lesions

**FIGURE 3:**
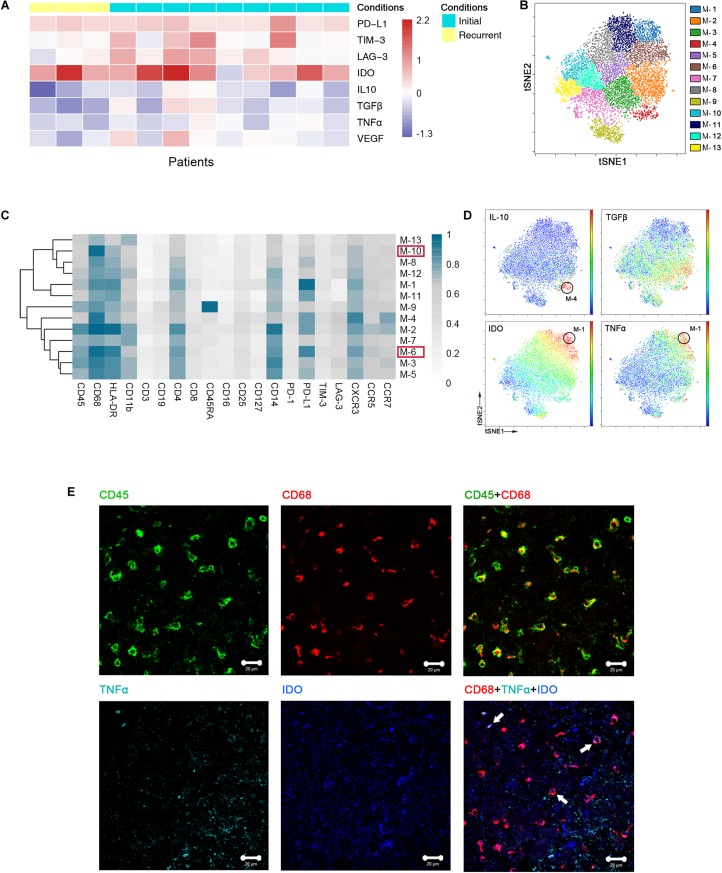
Heterogeneous characterization of GAM phenotypes in GBM. **(A)** Heatmap showing relative marker expression levels in 3 recurrent and 9 initial GBM cases. The relative marker expression levels were determined by the ratios of the indicated marker expression levels of GAMs at the tumor site to those of mononuclear phagocytes in pPBMCs. **(B)** ViSNE map, colored by clusters, displaying 13 GAM subgroups from a representative patient. **(C)** Heatmap showing the normalized expression of the indicated markers for 13 GAM clusters identified from the representative patient. **(D)** Normalized expression of the indicated markers on the viSNE map. **(E)** Representative GBM tissue stained for CD68 (red), CD45 (green), IDO (blue), and TNFα (cyan). Polychromatic immunofluorescence of CD45 and CD68 (upper) indicated that most CD45+ immunocytes in GBM were CD68+ cells. Co-staining of CD68, IDO, and TNFα (lower) demonstrated that GAMs could co-express TNFα and IDO (Arrows).

Previous studies showed the extensive infiltration of gliomas with microglia and peripheral macrophages (29), collectively termed GAMs. In the current study, GAMs were the most enriched population in GBM lesions. They showed inter-tumoral heterogeneity, as immune checkpoints PD-L1, lymphocyte activation gene-3 (LAG-3) and T cell immunoglobulin domain and mucin domain-3 (TIM-3), immunosuppressive cytokines interleukin-10 (IL-10) and transforming growth factor-β (TGFβ), tumor necrosis factor-α (TNFα) and vascular endothelial growth factor (VEGF) were expressed at various levels among patients (Figure 3A).

We performed a hierarchical cluster analysis of the GAM subpopulations using automatic cluster gate functionality to fully capture the heterogeneity of the GAM compartment. The GAMs were identified based on the expression of protein markers, including CD45, CD68, and CD11b, and then subdivided into 13 subgroups according to the surface markers (Figure 3B).

Regarding the expression of the immune checkpoint and cytokines among each subset, GAM phenotypes showed substantial intra-tumoral diversity. One group involving cluster M-6 displayed higher HLA-DR and CD68 expression levels and lower CD11b expression levels than the other groups, suggesting that these GAM cells were mature (29). This cluster was characterized by high expression of the immune checkpoint marker PD-L1. By expressing PD-L1 on the cell surface, GAMs may promote T cell apoptosis (30). Additionally, M-10 was also recognized in the mature group, but PD-L1 was expressed at lower levels than M-6 in this group (Supplementary Figure S1A). Among the GAM subsets, PD-L1 was frequently expressed, while certain immune checkpoints, such as TIM-3 and LAG-3, were seldom expressed (Figure 3C). At the single-cell level, surprisingly, the viSNE map showed that a certain GAM subgroup (M-1) could coexpress anti-tumor (TNFα) and pro-tumor (IDO) markers, while PD-L1 was also highly expressed in this subgroup (Figures 3C,D). We revealed that mononuclear macrophage infiltration in GBM lesions using H&E staining (Supplementary Figure S1B). Polychromatic immunofluorescence verified the finding that anti-tumor (TNFα) and pro-tumor (IDO) markers were co-expressed in certain GAM subgroups (Figure 3E).

We merged GAMs from all initial patients for analysis and found similar trends as the representative patient. GAMs can be divided into subgroups of different phenotypes (Supplementary Figure S1C). Anti-tumor and pro-tumor markers were shown to be co-expressed in certain subgroups (Supplementary Figures S1D,E).

### T Cells Are Exhausted and Tregs Are Increased in Initial GBM Lesions

**FIGURE 4:**
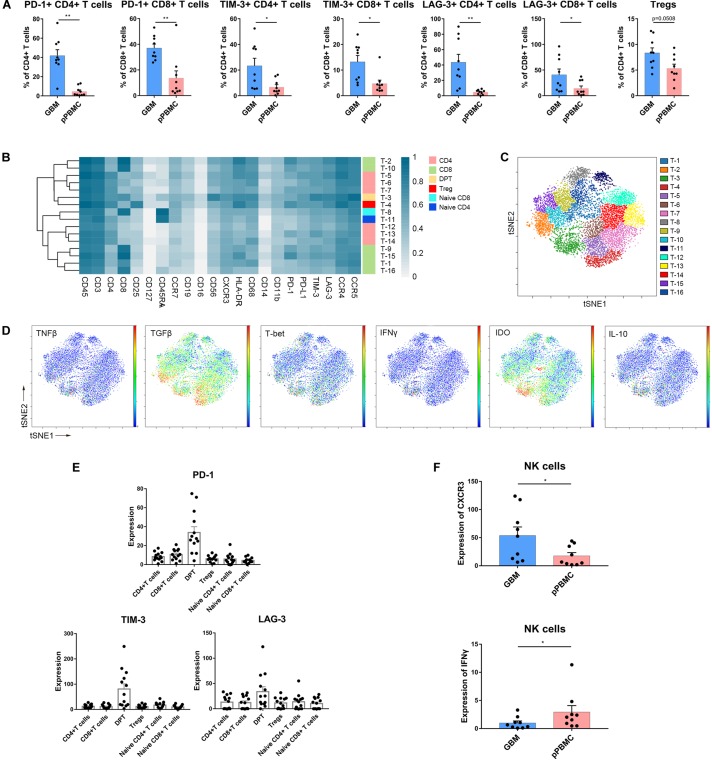
Exhausted T cell compartment in GBM. **(A)** Bar plots showing the frequencies of T cell subgroups across tumor sites and pPBMCs from patients with initial GBM (by Wilcoxon matched-pairs signed rank test). Bar plots show the mean with SEM (*p < 0.05, **p < 0.01). **(B)** Heatmap showing the normalized expression of markers for the 16 T cell clusters identified from a representative patient. **(C)** ViSNE map, colored by clusters, displaying T cell subgroups from the representative patient. **(D)** Normalized expression of the indicated markers on tumor T cells shown by viSNE plot. **(E)** Bar pots of PD-1, LAG-3, and TIM-3 expression in T cell subsets across all patients with initial GBM. Bar plots show the mean with SEM. **(F)** Bar plots demonstrating CXCR3 and IFNγ expression in NK cells across tissue samples from initial GBM patients and the paired pPBMCs (by the Wilcoxon matched-pairs signed rank test). Bar plots show the mean with SEM (*p < 0.05).

Specifically, compared to those in pPBMCs, the Treg proportions in the tumor lesions were significantly increased across all patients (*p* = 0.0508) (Figure 4A and Supplementary Figure S2A). PD-1+, TIM-3+ or LAG-3+ T cells are recognized as exhausted subsets (31, 32). Compared to that in pPBMCs, the proportions of exhausted CD4+ and CD8+ T cells were distinctly higher at the tumor sites (Figure 4A).

According to the surface markers, the T cells were subdivided into 16 subgroups. The expression profiles of the T cell clusters were visualized in a heatmap (Figure 4B), and heterogeneity in the immune-related marker levels was assessed at the single-cell level using viSNE maps (Figure 4C). This approach led to the identification of six CD4+ phenotypes, six CD8+ phenotypes, one CD4+/CD8+ double-positive phenotype, one naïve CD4+ phenotype, one naïve CD8+ phenotype and one Treg phenotype.

Several studies have shown that CD4+/CD8+ double-positive T cells (DPTs) are more than just a developmental stage (33). At the single-cell level, surprisingly, the viSNE map showed that in initial GBMs, DPTs were the major source of IL-10, IDO and TGFβ (Figure 4D). Importantly, the DPTs at the tumor sites expressed higher levels of PD-1, LAG-3, and TIM-3 than the CD4+ T cells, CD8+ T cells, Tregs, and naïve T cells (Figure 4E).

### NK Cells Are Not Cytolytic in GBM Lesions

Strikingly, NK cell proportions were not significantly increased at the tumor site compared with those in the peripheral blood of patients. The NK cells that infiltrated into the tumor lesions expressed higher levels of CXC chemokine receptor 3 (CXCR3) (*p* < 0.05) (Figure 4F), a molecule reported to be required for NK cell infiltration (34), than those in peripheral blood. However, the NK cells that remained at the tumor site were no longer cytolytic, as these cells expressed lower levels of interferon-γ (IFNγ) (*p* < 0.05) (Figure 4F). Moreover, NK cells infiltrated into recurrent GBMs presented similar characteristics (Supplementary Figures S2B,C).

### Recurrent GBMs Share Similar Immune Signatures With Initial GBMs

**FIGURE 5:**
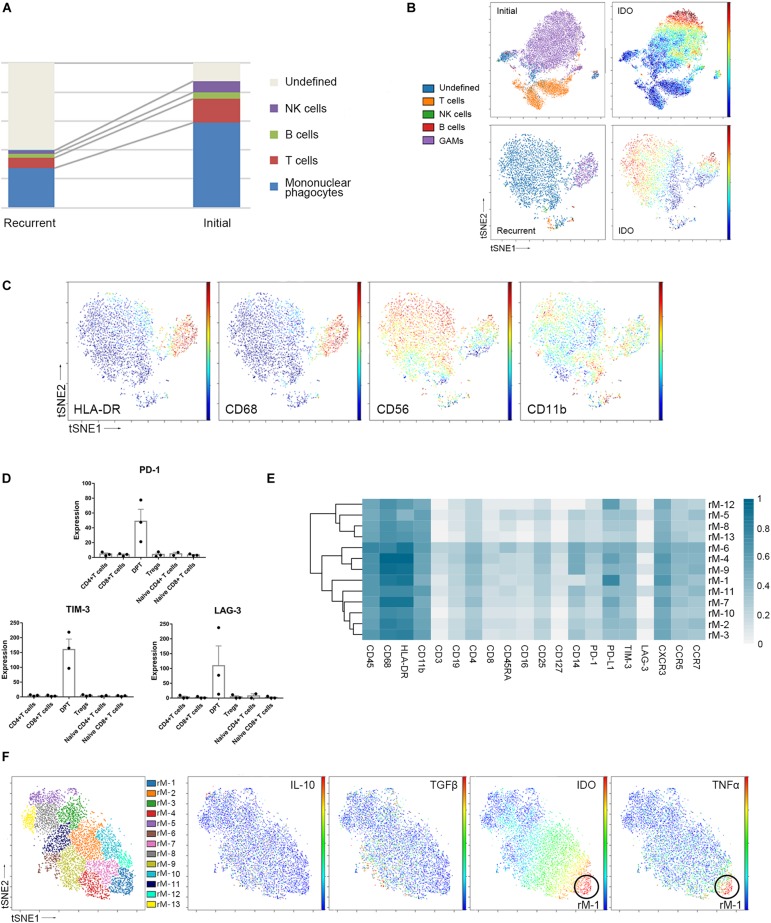
Recurrent and initial GBMs share similar immune signatures. **(A)** The frequencies of recurrent and initial GBM immunocytes. Composition of the CD45+ compartment showing the average frequencies of major immune lineages for each tissue. **(B)** ViSNE maps of representative patients with initial and recurrent GBM, colored by immunocyte subsets (left), displaying the expression level of IDO in undefined CD45+ cells (right). **(C)** ViSNE maps from the representative recurrent patient displaying expression levels of the indicated markers in undefined CD45+ cells. **(D)** Bar pots of PD-1, LAG-3 and TIM-3 expression in T cell subsets across all patients with recurrent GBM. Bar plots show the mean with SEM. **(E)** Heatmap showing the normalized expression of markers from the panel of 13 GAM clusters identified from a representative recurrent patient. **(F)** ViSNE map, colored by clusters, displaying GAM subgroups and the normalized expression of the indicated markers from the representative recurrent patient.

Compared to that in initial GBM tissues, the proportion of GAMs in the recurrent GBMs was decreased (59.05% vs. 27.87%, *p* < 0.05). Furthermore, the proportion of undefined CD45+ immune cells was changed (5.18% vs. 58.26%, *p* < 0.05). The undefined immune cells were regarded as CD45+ infiltrating immune cells but could not be defined as specific immune cells by the present panel which included 30 antibodies. Concluding that the number of these cells expanded is difficult because their identification was not possible. The proportions of the immunocyte subgroups (T cells, B cells, and NK cells) in the recurrent GBM samples were similar to those in the initial GBM samples (Figure 5A and Supplementary Figure S3A).

The undefined CD45+ cells in the recurrent GBMs expressed higher levels of the immune checkpoint protein IDO than those in the initial GBMs (Figure 5B). In the viSNE map of recurrent GBM, the undefined immune cells, which were located close to or mixed with GAMs, expressed IDO, CD56, and CD11b at levels similar to those in GAMs but expressed less HLA-DR than GAMs (Figures 5B,C).

Compared with the initial GBMs, the recurrent GBMs displayed similar suppressive immune changes. Exhausted T cell proportions were not significantly between the initial and recurrent GBMs (Supplementary Figure S3B). DPTs in the recurrent GBMs also expressed higher levels of the immune checkpoint proteins PD-1, LAG-3, and TIM-3 than the CD4+ T cell, CD8+ T cell, naïve T cell, and Treg subgroups (Figure 5D).

Glioma-associated microglia/macrophages in the recurrent GBM were subdivided into 13 subgroups based on the surface markers (Figure 5E). Similar to those in initial GBMs, one of the GAM subpopulations (rM-1) could co-express anti-tumor (TNFα) and pro-tumor (IDO) markers, while PD-L1 was also highly expressed in the recurrent GAMs (Figures 5E,F).

## Discussion

The GBM immune microenvironment plays a critical role in tumor development, progression, and prognosis. A comprehensive understanding of the intricate milieu and its interactions remains unclear, and single-cell analysis is crucially needed. With CyTOF approach, we aimed to analyze infiltrating immune cells from initial and recurrent GBM surgical tissues, both of which were coupled with their paired pPBMCs. Using a panel of 30 markers, we provide a single-cell view of the complex GBM immune microenvironment. Our study confirmed that GAMs, as the dominant infiltrating immune cell population, exhibit substantial inter- and intra-tumoral heterogeneity in the GBM immune microenvironment (Figures 2, 3), and increased proportions of exhausted T cell subpopulations and Tregs substantially contribute to local immune suppressive characteristics (Figure 4). Recurrent and initial GBMs were shown to share similar immune signatures except that the proportion of GAMs was decreased in the recurrent GBM samples compared with the initial GBM samples (Figure 5 and Supplementary Figure S3).

As the largest intra-tumoral immune cell population, GAMs interact with tumor cells, express a variety of immunosuppressive cytokines and play an emerging role in tumor progression and the regulation of anti-tumor immune responses (35, 36). Taking advantage of CyTOF technology, we used surface markers to demonstrate inter- and intra-tumoral heterogeneity in the GAM population, which was also shown to play diverse roles in gliomagenesis, as immune checkpoints, immunosuppressive cytokines, TNFα and VEGF were differentially expressed in the GAMs among the patients (Figure 3A). Meanwhile, GAM subpopulations showed different phenotypic patterns presenting different predominant immune checkpoints and immunosuppressive cytokines (Figures 3B,C). Moreover, anti-tumor cytokines and immunosuppressive cytokines or immune checkpoints may be expressed simultaneously in the same GAM subpopulation (Figures 3D,E). Substantial diversity and specificity are characteristic of GAMs, and dissecting the heterogeneity and specific roles of each intra-tumoral GAM subset may be of critical importance for successfully targeting the immunosuppressive GAM population in a clinical setting and for the individual design of future immunotherapies (37).

CD8+ T cells that are specific for tumor-associated antigens can engage tumor cells in an antigen-specific manner, and these cells drive anti-tumor immunity by secreting effector cytokines, releasing cytotoxic molecules and inducing apoptosis in tumor cells (38). In addition to CD8+ T cells, IFNγ-expressing CD4+ T cells, and NK cells have potent anti-tumor effects in the immune microenvironment (34). The infiltration of tumors by T cells is generally interpreted as a sign of immune recognition, and there is a growing effort to reactivate dysfunctional T cells at such tumor sites (39). In our study, we found that T cell populations exhibited complex diversity based on their surface markers. The markers PD-1, LAG-3, TIM-3, and IDO are highly expressed in some T cell subgroups. Immune checkpoint (PD-1, LAG-3 and TIM-3)-positive CD4+ and CD8+ T cells cannot exert an anti-tumor effect and are regarded as nonfunctional or exhausted subsets. Although the GBM microenvironment was infiltrated with CD4+ T cells, CD8+ T cells, and NK cells, the proportions of nonfunctional immune cell subpopulations and Tregs increased, while whole T cell numbers were reduced at the tumor site. DPTs at tumor sites secrete more IL-10, IDO, and TGFβ and express higher levels of immune checkpoints PD-1, LAG-3, and TIM-3 than Tregs, CD4+ T cells and CD8+ T cells. However, they also secrete T-bet and TNFβ; thus, the DPTs in the context of the GBM microenvironment might play a dual role in the immune response. Tregs inhibit the proliferation of any cytokine-secreting effector T cells and are potent suppressors of the adaptive immune response (40). NK cells showed a high infiltration ability in GBM lesions, but they did not show a strong cytolytic ability according to their surface receptors and secreted cytokines. Our results indicated that the intrinsic capacity of intra-tumoral effector T cells and NK cells was impaired, which suggests that in addition to increasing their quantity, approaches that simultaneously promote the anti-tumor quality and eliminate the pro-tumor ability of these cells will benefit clinical efforts to reactivate intra-tumoral immune cells.

Little is known about how the microenvironment changes in recurrent GBM. Only 20–30% of recurrent GBM cases are accessible for surgical treatment (41). Even less is known about changes in the immune environment and the immunogenicity of recurrent tumors (42). Mohme et al. (43) used flow cytometry and cytokine assays to profile tumor-infiltrating lymphocytes and blood lymphocytes from GBM patients. The study showed that the tumor-infiltrating lymphocytes of recurrent GBMs exhibited restricted T cell receptor repertoire clonality and a more activated memory phenotype than those of initial GBMs (43). Using CyTOF method, the current study demonstrated that on a single-cell basis, the recurrent and initial GBMs shared similar immune signatures; however, the proportion of GAMs in the recurrent GBMs was decreased compared with that in initial GBMs (Supplementary Figure S3). Interestingly, in the recurrent GBM samples, the proportion of undefined CD45+ immune cells was significantly increased and overwhelmed the proportion of GAMs (Figure 5A). Furthermore, the undefined immune cell subset in recurrent GBM might partially functionally resemble GAMs in its presentation of strong immunosuppressive features, but more research is needed on this topic. Because most immunotherapies are first applied to recurrent GBMs, comparing the immune landscapes of initial and recurrent GBMs will help predict the efficiency of immunotherapy applications in treatment-naïve GBM patients.

Our study has some limitations. Deciphering the immune environment of GBMs and determining the association between the GBM immune microenvironment and patient prognosis requires more cases. We acknowledge that the small number of recurrence cases may result in a lack of sufficient power to identify immune microenvironment differences between initial and recurrent cases. We performed clustering analysis to study immune environment heterogeneity. We must admit that in our study, clustering in CyTOF analyses can change depending on the parameters selected, and the number of clusters is not absolute. Some of our findings should be further explored by single-cell RNA sequencing, particularly regarding the changes in undefined immune cells in recurrent GBM. Ideally, dissecting the features of recurrent GBM should incorporate paired samples and their corresponding initial and recurrent GBM samples from a single patient.

## Data Availability Statement

The data that support the findings of this study are available from the corresponding author upon reasonable request.

## Ethics Statement

This research was approved by the Institutional Review Board (IRB) and Ethics Committee of Beijing Tiantan Hospital (Beijing, China). Written informed consent was obtained from all patients and healthy donors.

## Author Contributions

YC, JZ, DC, JaW, and SW conceived and designed the study. WF and WW analyzed and interpreted the data. HL, YJ, RH, JeW, and ZY participated in sample collection and data acquisition. All authors participated in drafting the manuscript, read and approved the final version of the manuscript, and gave their consent for publication.

## Conflict of Interest

The authors declare that the research was conducted in the absence of any commercial or financial relationships that could be construed as a potential conflict of interest.

## Footnotes

1 www.cytobank.org
