# Supplementary material for: Single-Cell Atlas Reveals Complexity of the Immunosuppressive Microenvironment of Initial and Recurrent Glioblastoma
Source: Front Immunol. 2020 May 7;11:835. doi: 10.3389/fimmu.2020.00835 (PMC7221162; doi:10.3389/fimmu.2020.00835)
Supplement: Supplementary file 1 [file Table_1.docx]

**Table S1 Mass cytometry panel**

| **Marker** | **Metal isotope** | **Source** | **Identifier** | **Description** |
| --- | --- | --- | --- | --- |
| CD45 | 89Y | Fluidigm | Cat#3089003B | Hematopoietic marker |
| CD3 | 149Sm | Biolegend | Cat#300502 | T cells |
| CD19 | 150Sm | Biolegend | Cat#302247 | B cells |
| CD4 | 145Nd | Fluidigm | Cat#3145001B | CD4 T cells |
| CD8 | 146Nd | Fluidigm | Cat#3146001B | CD8 T cells |
| CD11b | 209Bi | Fluidigm | Cat#3209003B | Myeloid marker |
| CD68 | 171Yb | Fluidigm | Cat#3171011B | Glycoprotein |
| CD14 | 169Tm | Biolegend | Cat#301802 | Monocyte marker |
| CD16 | 148Sm | Fluidigm | Cat#3148004B | NK cells |
| CD56 | 155Gd | Fluidigm | Cat#3155008B | NK cells |
| CD25 | 147Sm | Biolegend | Cat#302602 | Tregs |
| CD127 | 176Yb | Fluidigm | Cat#3176004B | Tregs |
| CD45RA | 143Nd | Fluidigm | Cat#3143006B | Naïve T cells |
| CCR5 | 144Nd | Fluidigm | Cat#3144007A | Chemokine receptor |
| CCR7 | 159Tb | Fluidigm | Cat#3159003A | Naïve T cells |
| HLA-DR | 174Yb | Fluidigm | Cat#3174001B | MHC class II molecule |
| CCR4 | 158Gd | Fluidigm | Cat#3158032A | Chemokine receptor |
| CXCR3 | 141Pr | Biolegend | Cat#353733 | Chemokine receptor |
| PD-1 | 151Eu | Biolegend | Cat#329941 | Checkpoint receptor |
| TIM-3 | 153Eu | Fluidigm | Cat#3153008B | Checkpoint receptor |
| LAG-3 | 175Lu | Biolegend | Cat#369302 | Checkpoint receptor |
| PD-L1 | 154Gd | Biolegend | Cat#329719 | Checkpoint ligand |
| TNFα | 152Gd | Biolegend | Cat#502902 | Cytokine |
| TNFβ | 172Yb | Biolegend | Cat#503002 | Cytokine |
| IFNγ | 168Er | Fluidigm | Cat#3168005B | Cytokine |
| T-bet | 161Dy | Biolegend | Cat#644802 | Transcription factor |
| VEGF | 162Dy | Biolegend | Cat#627501 | Cytokine |
| IDO | 160Dy | Biolegend | Cat#695002 | Immune checkpoint protein |
| TGFβ | 163Dy | Fluidigm | Cat#3163010B | Immunosuppressive cytokine |
| IL-10 | 166Er | Fluidigm | Cat#3166008B | Immunosuppressive cytokine |
