# Supplementary material for: Single-Cell Atlas Reveals Complexity of the Immunosuppressive Microenvironment of Initial and Recurrent Glioblastoma
Source: Front Immunol. 2020 May 7;11:835. doi: 10.3389/fimmu.2020.00835 (PMC7221162; doi:10.3389/fimmu.2020.00835)
Supplement: Supplementary file 2 [file Table_2.docx]

**Table S2 Cell type identification**

| **Immunocyte** | **Markers** |
| --- | --- |
| T cell | CD45+ CD3+ |
| CD4+ T cell | CD45+ CD3+ CD4+ |
| CD8+ T cell | CD45+ CD3+ CD8+ |
| B cell | CD45+ CD19+ |
| NK cell | CD45+ CD3- CD16+ CD56+ |
| Monocyte | CD45+ CD14+CD16+ |
| Macrophages/Microglia | CD45+ CD11b+ CD68+ |
| Treg | CD45+ CD4+ CD25+ CD127- |
| Naïve CD4+ T cell | CD45+ CD45RA+ CCR7+ CD4+ |
| Naïve CD8+ T cell | CD45+ CD45RA+ CCR7+ CD8+ |
