# Supplementary material for: Single-Cell Atlas Reveals Complexity of the Immunosuppressive Microenvironment of Initial and Recurrent Glioblastoma
Source: Front Immunol. 2020 May 7;11:835. doi: 10.3389/fimmu.2020.00835 (PMC7221162; doi:10.3389/fimmu.2020.00835)
Supplement: Supplementary file 3 [file Table_3.docx]

Supplementary Material

**Supplementary Figure 1.** **Heterogeneous characterization of GAM phenotypes identified from all patients.**

**(A)**. Bar plots demonstrating the PD-L1 expression levels in M-6 and M-10 (by the Mann-Whitney test). Each dot represents one cell; there are 844 cells in the M-6 group and 623 cells in the M-10 group. Bar plots show the mean with SEM (****p<0.0001). **(B).** H&E staining (×400) showing infiltration of mononuclear macrophages in GBM lesions (Arrows). **(C)**. ViSNE map, colored by clusters, displaying 13 GAM subgroups from all 13 initial patients. A total of 1000 GAM cells per patient were used for analysis. **(D)**. Heatmap showing the normalized expression of markers from the panel of 13 GAM clusters identified from all initial patients. **(E)**. Normalized expression of the indicated markers on the viSNE map from all patients. Antitumor and pro-tumor markers were co-expressed in certain GAM subgroup (M-d).

**Supplementary Figure 2. Cytolytic NK cells are dysregulated at the tumor site.**

**(A)**. Bar plots showing the frequencies of T cell subgroups across tumor sites and pPBMCs from patients with initial GBM (by Wilcoxon matched-pairs signed rank test). Bar plots show the mean with SEM (NS, no significance). **(B** and **C)**. Bar plots displaying CXCR4 and IFNγ expression in NK cells across tissue samples from patients with initial and recurrent GBM (by the Mann-Whitney test). Bar plots show the mean with SEM (NS, no significance).

**Supplementary Figure 3. Immunosuppressive changes in the recurrent GBM microenvironment.**

**(A)**. Bar plots showing the frequencies of immune cell subgroups for each patient with initial and recurrent GBM (by the Mann-Whitney test). Error bars indicate the SEM (NS, no significance; *p < 0.05 and **p < 0.01). **(B)**. Bar plots showing the frequencies of T cell subgroups across patients with initial and recurrent GBM (by the Mann-Whitney test). Bar plots show the mean with SEM (NS, no significance).

**Supplementary Table 1.** Mass cytometry panel.

**Supplementary Table 2.** Cell type identification.
