# Supplementary figures and images for: Single-Cell Atlas Reveals Complexity of the Immunosuppressive Microenvironment of Initial and Recurrent Glioblastoma
Source: Front Immunol. 2020 May 7;11:835. doi: 10.3389/fimmu.2020.00835 (PMC7221162; doi:10.3389/fimmu.2020.00835)

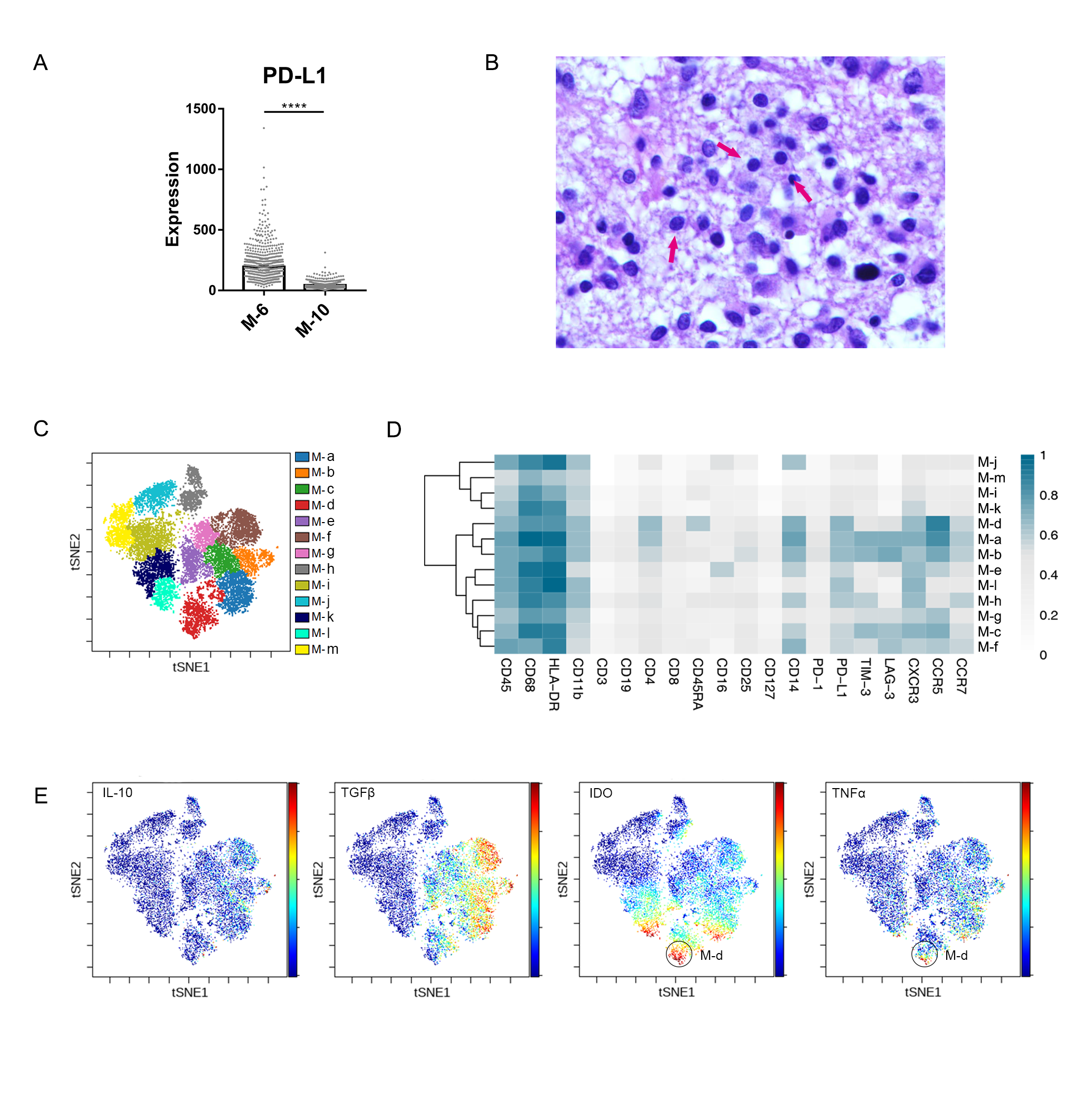

Supplement: Supplementary file 4 [file Image_1.tif]

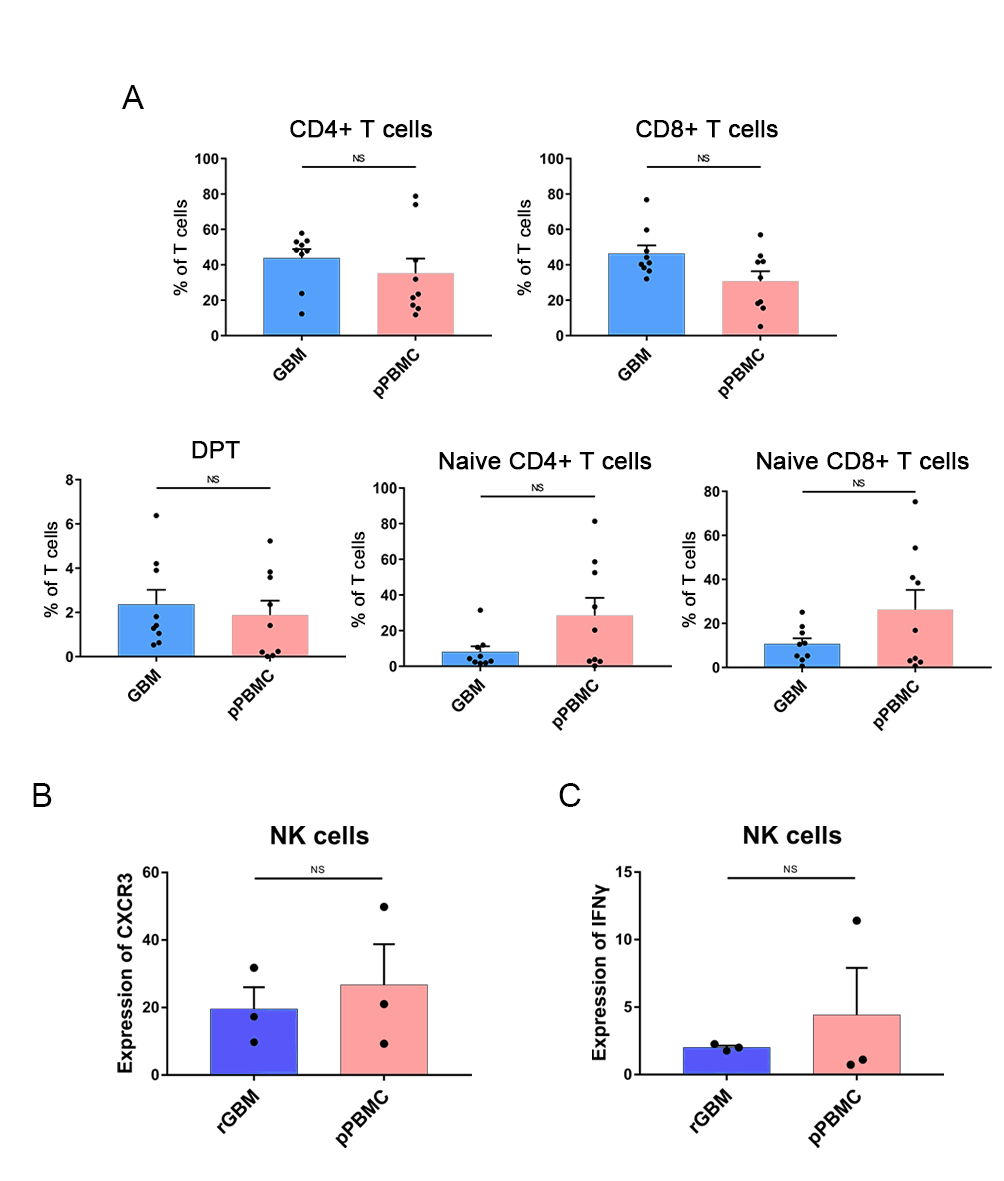

Supplement: Supplementary file 5 [file Image_2.tif]

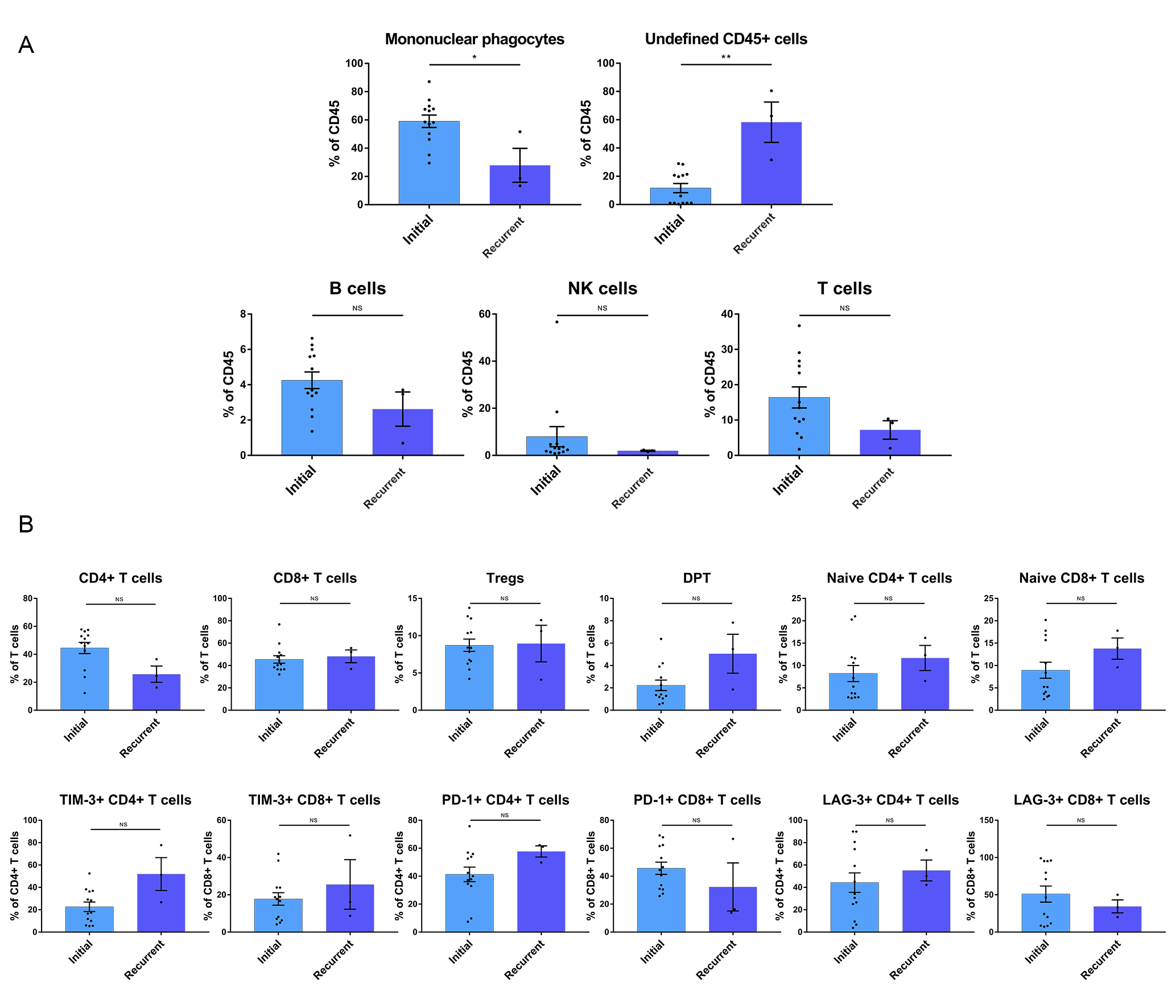

Supplement: Supplementary file 6 [file Image_3.tif]
